# Constructing Bi-Continuous Poly(urethane-*co*-amide) Networks from Hydroxylated Oleic Acid via Dynamic Self-Vulcanization for Super-Toughened Polylactic Acid Blends

**DOI:** 10.3390/polym18161981

**Published:** 2026-08-14

**Authors:** Dongmei Xie, Xiaodi Mao, Hongyu Li, Xudong Chen, Yuting Li, Hongzhi Liu

**Affiliations:** School of Materials Science and Engineering, NingboTech University, Ningbo 315100, China

**Keywords:** polylactic acid, dynamic vulcanization, bifunctional monomers, toughening

## Abstract

To demonstrate the applicability of the “dynamic self-vulcanization of bifunctional monomers” strategy for toughening polylactic acid (PLA), hydroxylated oleic acid (HOA) was synthesized via UV-initiated thiol–ene click chemistry, using oleic acid as the starting material. In the presence of an excess molar quantity of hexamethylene diisocyanate (HDI), the dynamic self-vulcanization of bifunctional monomers was employed to design PLA blends featuring extraordinary impact toughness. During the one-pot melt compounding, in situ formation and self-crosslinking of flexible poly(urethane-*co*-amide) (HPUA) toughening phase, together with its reactive compatibilization with the PLA matrix, were simultaneously accomplished. The aggregation of the HPUA domains enabled the morphological transformation of the PLA blend from a sea-island structure to a partially or fully bi-continuous one. At HPUA contents of 20 wt% or higher, the blend exhibited a bi-continuous morphology with a crosslinked HPUA network, attaining a notched impact strength exceeding 110 kJ/m^2^ and an elongation at break above 200%. In particular, when the HPUA content reached 20 wt%, the resulting PLA blend exhibited optimal impact toughness, with a notched IS of 132.1 kJ/m^2^ (30.7 times that of neat PLA). The primary toughening mechanism was determined to be the internal cavitation of the HPUA domains, which subsequently initiates the yielding of the surrounding PLA matrix. This study proposes an applicable and facile method for fabricating polymer materials that possess excellent impact toughness.

## 1. Introduction

As a bio-based aliphatic thermoplastic polyester, polylactic acid (PLA) is considered a prime contender among biodegradable and renewable polymers for substituting traditional petroleum-based materials. This is due to its remarkable mechanical properties, good processability, excellent biocompatibility, and biodegradability [1,2,3]. Nevertheless, the intrinsic brittleness of PLA, characterized by inferior impact strength ranging from 1.9 to 4.3 kJ/m^2^, significantly restricts its extensive application in some fields that demand high impact toughness [4,5,6], which originates from the low-chain-segment mobility at ambient temperature (*T*_g_ ≈ 55–60 °C) and the tendency toward unstable craze propagation rather than energy-dissipating shear yielding or cavitation [7,8].

Owing to the limited compatibility between most polymer-toughening agents and the PLA matrix [9], it is of great importance to accurately regulate interfacial compatibility and phase morphology in order to achieve super-tough PLA (impact strength above 530 J/m) [10]. Consequently, many strategies have been developed to improve the interfacial compatibility between the PLA matrix and the toughening phase [11,12,13,14,15]. Among them, dynamic vulcanization has been widely recognized as one of the most efficient and practically feasible [16]. Distinct from conventional rubber-toughened PLA blend systems that rely solely on reactive compatibilization, dynamic vulcanization can not only generate a crosslinked toughening phase but also facilitates interfacial compatibilization without the need for any additional compatibilizer. Specifically, in situ crosslinking during dynamic vulcanization enhances the melt viscosity and elasticity of the dispersed phase, enabling its morphological evolution from a sea-island to a bi-continuous structure under appropriate composition ratios and processing conditions [17,18]. The resulting continuous network provides abundant stress-concentration and cavitation sites, effectively promoting energy dissipation within the system. Under impact loading, the crosslinked rubbery domains undergo internal cavitation, which further triggers shear yielding of the PLA matrix—this constitutes the primary energy dissipation mechanism [7,8]. In contrast, in reactive blending systems where only interfacial compatibilization occurs, the dispersed phase does not form a crosslinked network, resulting in limited phase morphology control. The toughening process relies primarily on interfacial delamination, which hinders the extensive plastic yielding of the matrix via internal cavitation. Numerous petroleum-based and bio-based elastomers or rubbers, such as thermoplastic polyurethane [2,19], polyesters [20,21,22,23], natural rubber (NR) [24,25,26,27], epoxidized natural rubber (ENR) [28,29,30,31], nitrile butadiene rubber (NBR) [32,33], and ethylene-*co*-vinyl acetate (EVA) [34], have been successfully utilized to achieve a superior toughening effect on PLA in terms of dynamic vulcanization.

Compared to crosslinkable elastomers or rubbers, reactive monomers or prepolymers capable of rapidly forming a crosslinked elastomer during melt blending have become more attractive precursors for toughening PLA, owing to their superior design flexibility, higher reactivity, and lower initial viscosity. During dynamic vulcanization, reactive monomers or prepolymers can simultaneously undergo polymerization, crosslinking, and reactive compatibilization. Consequently, the phase morphology of the toughening phase can be rationally controlled by varying the functional group molar ratio of the toughening monomers. To maintain the bio-based characteristic of PLA materials, several bio-based monomers, such as epoxidized soybean oil (ESO) [35,36], castor oil (CO) [17,37], and plant oil-derived dimer acid [18,38], have been utilized in dynamic vulcanization.

Given that dynamic vulcanization needs to be accomplished quickly and completely within the brief melt-blending process, highly reactive isocyanate components have been widely used to enhance the toughness of PLA. Zeng et al. [39] prepared a super-toughened PLA/CPU binary blend with a notched impact strength of 546 J/m by melt-blending polyethylene glycol (PEG) as the diol component with PLA and polymeric methylene diphenylene (PMDI) (functionality 2.6). In the melt blending process, the CPU phase forms through in situ polymerization of PEG and PMDI, while the reaction between NCO groups and terminal hydroxyl groups of PLA promotes interfacial compatibility between PLA and CPU. In subsequent investigations, Hadadoui et al. [40] fabricated bio-based PLA/PU blends through dynamic vulcanization of a fatty acid-derived polyester polyol with hexamethylene diisocyanate in the presence of PLA, using glycerol as a crosslinker. As the crosslinked polyurethane (PU) phase formed within the PLA matrix, the impact strength of the blend increased significantly. Although these studies demonstrated that in situ formed crosslinked polyurethane (CPU) elastomers could effectively improve the impact toughness of PLA, the tensile strength of the resulting blends typically suffered a substantial loss, retaining only ~37–47% of that of neat PLA [1,17,23,37,39,41]. Moreover, in typical PLA/polyisocyanate/polyol systems, the toughening phase is generally prepared from either diols with polyisocyanates or polyols with diisocyanates, while maintaining a fixed molar ratio of isocyanate to hydroxyl groups (n_NCO_/n_OH_) of 1:1. Our research group recently reported a strategy for toughening PLA via “dynamic self-vulcanization of dual difunctional monomers”. This work describes the fabrication of a fully bio-based super-tough PLA blend by employing an excess of bifunctional L-lysine diisocyanate (LDI) to induce the dynamic vulcanization of hydrogenated dimer acid (HDA) [18]. By manipulating the NCO/COOH equivalent ratio between LDI and HDA, the notched impact strength reached as high as 109.8 kJ/m^2^, while the tensile strength and modulus of the blends were well retained [38]. Furthermore, dynamic vulcanization of PLA with plant-based hydrogenated diol (Prilop) was attempted using an excess of HDI as a reactive crosslinker. By adjusting the equivalent ratio of functional groups (n_NCO_/n_OH_) between these two bifunctional monomers, the notched impact strength (IS) and phase morphology of the blend could be tuned over a wide range [41].

To demonstrate the applicability of this dynamic vulcanization strategy, in this study, hydroxylated oleic acid (HOA) was synthesized from oleic acid via a solvent-free thiol–ene click reaction. Subsequently, HOA was dynamically vulcanized with PLA in the presence of excessive HDI to prepare a PLA blend. During the melt-blending process, HOA undergoes in situ polymerization with HDI to form a soft crosslinked poly(urethane-*co*-amide) elastomer (HPUA). Concurrently, the excess isocyanate groups within the HPUA domains might participate in reactive compatibilization with the PLA matrix, strengthening the interfacial adhesion between the two phases and thus significantly enhancing the impact toughness of the resulting PLA blend. Consequently, the effects of HPUA content on mechanical and dynamic rheological behaviors, as well as the morphological development of the resulting PLA blends, were systematically examined. The reaction and impact-toughening mechanisms were also elucidated.

## 2. Experimental Section

### 2.1. Materials

Polylactide (PLA 4032D) was supplied by NatureWorks LLC. (Plymouth, MN, USA). Oleic acid (OA, 85%), 2-mercaptoethanol (ME, 99%), 2,2-dimethoxy-2-phenylacetophenone (DMPA, 99%), and hexamethylene diisocyanate (HDI, 99%) were obtained from Aladdin Company (Shanghai, China). Ethyl acetate, anhydrous magnesium sulfate, sodium chloride, and chloroform were acquired from Guoyao Chemical (Shanghai, China).

### 2.2. Preparation of Hydroxylated Oleic Acid

Under solvent-free conditions, oleic acid (OA) (13.3 g, 40 mmol) and 2-mercaptoethanol (9.5 g, 120 mmol) were placed in a 100 mL quartz tube, with a molar ratio of double bonds to thiol groups of 1:3. Subsequently, 2,2-dimethoxy-2-phenylacetophenone (DMPA) (0.31 g, 3.0 equiv. relative to OA) was added as an initiator. The mixture was irradiated with a 50 W, 365 nm UV lamp for 4 h while being continuously stirred in a dark chamber. Afterwards, the resulting product was diluted with ethyl acetate and then washed multiple times with saturated sodium chloride solution to eliminate residual ME and any disulfide by-products. The obtained organic layer was dried over anhydrous MgSO_4_, followed by filtration. Finally, after removing organic solvents using rotary evaporation, the hydroxyoleic acid (HOA) was obtained as a lightly yellow viscous liquid consisting of a mixture of regioisomers, with a yield of 88%.

^1^H NMR (400 MHz, DMSO-d_6_, δ, ppm): 0.86 (t, —C***H***_3_), 2.18 (t, —C***H***_2_—C(O)—, oleic acid chain), 3.47 (t, —C***H***_2_—OH), 2.59 (m, —C***H***—S—), 4.75 (s, —O***H***), 11.97 (s, —COO***H***). ^13^C NMR (100 MHz, DMSO-d6, 298 K): 174.6 (—***C***OOH), 62.0 (—***C***H_2_—OH), 45.8 (—***C***H—S—), 14.1 (—***C***H_3_). FTIR (ATR; cm^−1^): 3478 cm^−1^ (OH), 2922 (CH_2_ aliphatic), 1705 (—C=O acid).

### 2.3. Preparation Sample

PLA pellets were dried in a vacuum oven at 75 °C for 12 h before melt-compounding. An XSS-300 torque rheometer (Shanghai Kechuang Rubber & Plastic Equipment Co., Ltd., Shanghai, China), equipped with two counter-rotating rotors, was employed for melt-compounding at 170 °C. Initially, a predetermined amount of dried PLA pellets were added to the chamber at a rotor speed of 60 rpm until complete melting was attained, as indicated by the torque curve (Figure S5). Subsequently, HOA was injected and melt-blend continued for another 2 min. Immediately following the addition of HDI, the rotor speed was raised to 100 rpm to guarantee complete reaction. The reaction progress was monitored via the melt torque curve. Once the torque stabilized, the compounding was stopped, and the resulting material was promptly removed from the chamber and cut into small granules. For comparison, neat PLA samples were also prepared under the same conditions.

In preliminary experiments, the PLA content was maintained at 80 wt% for all blend formulations. The effect of the molar ratio of HDI isocyanate groups (–NCO) to HOA reactive groups (–COOH and –OH) (n_NCO,HDI_/n_COOH+OH,HOA_) on the mechanical properties of the PLA/HPUA blends was then explored. We observed that the resulting blend showed the best impact toughness at a ratio of 1.6:1 (Table S2 and Figure S1). Accordingly, the n_NCO,HDI_/n_COOH+OH,HOA_ ratio was fixed at 1.6:1. Subsequently, a series of PLA blends with combined weight fractions of HOA and HDI varying from 10 wt% to 40 wt% were prepared. The prepared PLA blends are named “**PLA/HPUAx**”, where “**x**” indicates the weight percentage of (HDI + HOA) in the total feedstock. All blend samples were hot-pressed at 180 °C under 20 MPa to obtain 1 mm thick sheets for tensile testing and 3 mm thick sheets for notched impact testing, respectively.

### 2.4. Nuclear Magnetic Resonance Spectroscopy

A Bruker NMR spectrometer (Bruker, Billerica, MA, USA) was used to record the ^1^H and ^13^C NMR spectra, with dimethyl sulfoxide (DMSO-d_6_) serving as the solvent.

### 2.5. Fourier Transform Infrared (FT-IR) Spectroscopy

FT-IR measurements of the PLA/HPUA blends were performed on a Nicolet iS50 spectrometer (Thermo Fisher Scientific, Waltham, MA, USA) in the wavenumber range of 4000–400 cm^−1^, using a resolution of 4 cm^−1^ and 32 scans.

### 2.6. Thermogravimetric Analysis (TGA)

Under a nitrogen atmosphere, thermogravimetric analysis (TGA) was carried out on a TGA Q50 (NETZSCH Instruments, Selb, Germany) at a heating rate of 10 °C/min over a temperature range of room temperature to 700 °C.

### 2.7. Determination of Gel Content

A known weight of PLA blend (*W*_1_) was enclosed in pre-weighed filter paper (*W*_0_) and extracted with excess chloroform (CHCl_3_) in a Soxhlet apparatus for 48 h. After extraction, the filter paper containing the insoluble residue was dried at 70 °C until a constant weight (*W*_2_) was achieved. The gel content of the blend was then calculated using the formula below:

(1) $$\mathrm{Gel}\;\mathrm{content}\;=\;\frac{W_{2}-W_{0}}{W_{1}}\;\times \;100\%$$

### 2.8. Mechanical Properties

An ED26.104 universal testing machine (i-test Equipment Co., Ltd., Ningbo, China) was employed for tensile testing at room temperature, with a crosshead speed of 5 mm/min. Dumbbell specimens (4 mm wide, 2 mm gauge length) were cut from 1 mm thick compression-molded plates. Notched Izod impact tests were conducted using a DWS-6069 pendulum impact tester (Jiangsu Davos Machinery Technology Co., Yangzhou, China) at room temperature. The impact specimens had dimensions of 63.5 mm × 12.5 mm × 3.2 mm and a notch depth of 2.5 mm. A minimum of five specimens were tested per sample, and the results are reported as averages.

### 2.9. Dynamic Mechanical Analysis (DMA)

Dynamic mechanical analysis (DMA) of the PLA/HPUA blends was performed using a DMA 850 (TA Instruments, New Castle, DE, USA) in single-cantilever mode at a frequency of 1 Hz. The measurement was carried out over a temperature range of −100 °C to 150 °C at a heating rate of 3 °C/min.

### 2.10. Dynamic Rheological Measurement

Dynamic frequency sweep measurements under small-amplitude oscillatory shear were performed on an ARES-G2 rotational rheometer (TA Instruments, New Castle, DE, USA) equipped with 25 mm parallel-plate geometry. The disk specimens (25 mm diameter, 1 mm thick) were tested at 180 °C, and all tests were carried out within the linear viscoelastic region.

### 2.11. Scanning Electron Microscopy (SEM)

The phase morphology of the PLA/HPUA blends was observed using a Hitachi Regulus 8100 scanning electron microscope (SEM; Hitachi, Tokyo, Japan). Specimens were cryo-fractured in liquid nitrogen, and the fracture surfaces were then sputter-coated with gold.

### 2.12. Transmission Electron Microscopy (TEM)

A JEM-1230 transmission electron microscope (JEOL Ltd., Tokyo, Japan) was employed to investigate the phase morphology of the PLA/HPUA blends. Cryo-ultrathin sections were prepared using an EM UC7 microtome (Leica, Wetzlar, Germany) and subsequently exposed to RuO_4_ vapor for staining.

### 2.13. Determination of Acid Value and Hydroxyl Value

The acid value of HOA was determined according to ASTM D1980-87 [42]. Briefly, approximately 1 g of HOA sample was dissolved in 75–100 mL of hot 95% ethyl alcohol in a 500 mL Erlenmeyer flask. Following the addition of 0.5 mL of phenolphthalein indicator solution (10 g/L), the solution was titrated immediately with 0.5 N KOH solution to the first pink color that persisted for 30 s. The acid value is calculated as follows:

Acid value = (*V* × *N* × 56.1)/*S* (2)

where *V* is the volume of KOH or NaOH solution used for titration (mL), *N* is the normality of the KOH or NaOH solution, and *S* is the sample weight (g).

The hydroxyl value of HOA was determined according to ASTM D1957-86 [43]. A 2 g HOA sample was weighed into a 250 mL Erlenmeyer flask, and 10 mL of pyridine–acetic anhydride solution (3:1 *v*/*v*, freshly prepared) was added. The flask was fitted with a reflux condenser and heated on a steam bath for 1 h. Then, 10 mL of distilled water was introduced through the condenser, and the heating was continued for an additional 20 min. After cooling, 25 mL of neutralized n-butyl alcohol and 1 mL of phenolphthalein indicator solution were added. The mixture was then titrated with 0.5 N alcoholic KOH solution until a faint pink endpoint was reached.

(3) $$\mathrm{Hydroxyl}\;\mathrm{value}=\frac{B+(\;\frac{SA}{C}\;)-V}{S}N\;\times \;56.1$$

where *B* is the KOH volume for the reagent blank titration (mL), *A* is the KOH volume for the acid value titration (mL), *C* is the sample weight for acid value determination (g), *V* is the KOH volume for the acetylated specimen titration (mL), *S* is the sample weight for acetylation (g), and *N* is the normality of KOH solution.

## 3. Results and Discussion

### 3.1. Chemical Characterization of HOA

**Figure 1:**
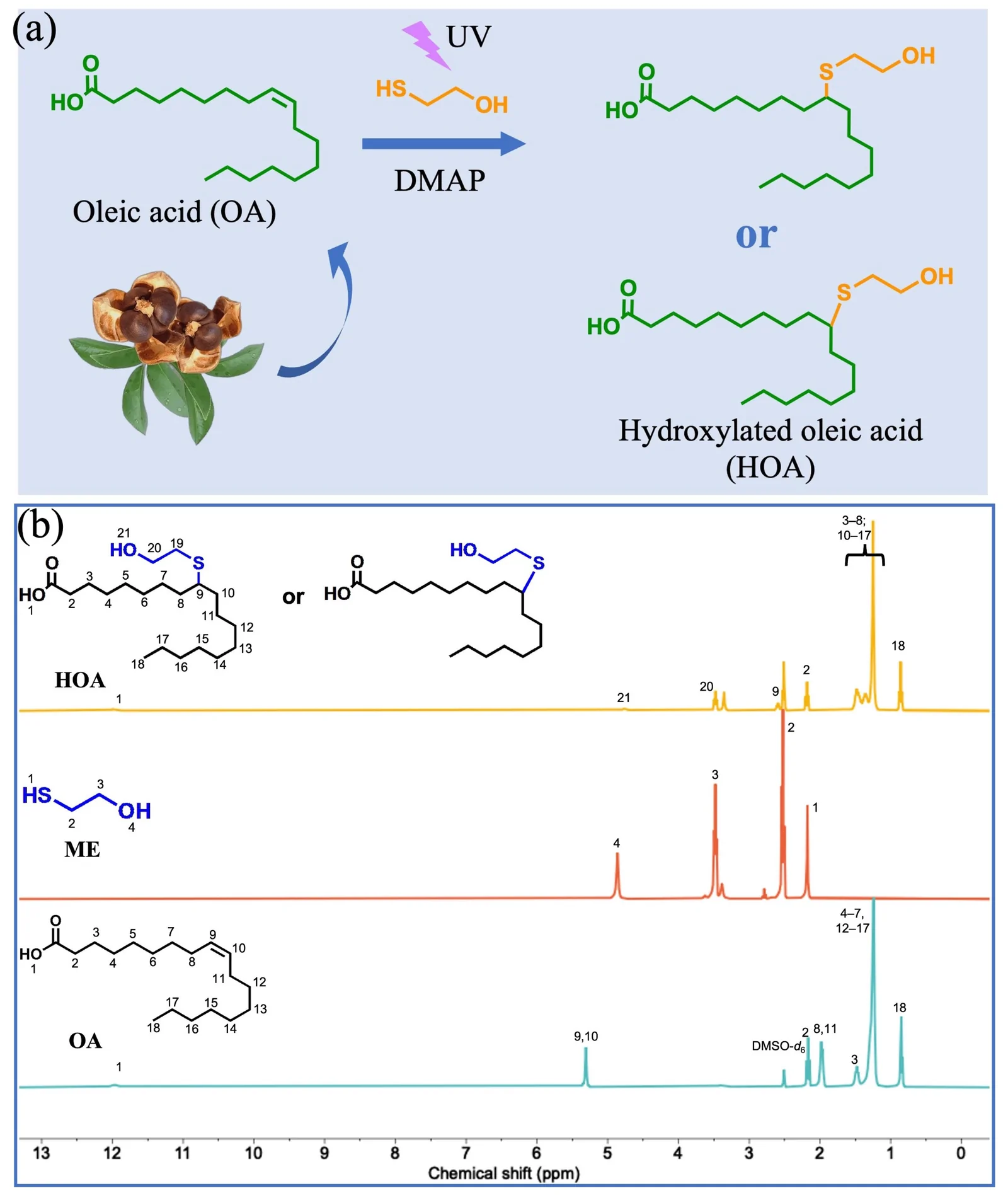
(**a**) Schematic of the synthesis of HOA by the “thiol−ene” click reaction, (**b**) ^1^H NMR spectra of OA, ME, and HOA. The numbers on the spectra correspond to the proton positions labeled on the respective molecular structures.

Using oleic acid (OA) as the starting material, hydroxyoleic acid (HOA) was prepared at high yield under solvent-free conditions through thiol–ene click chemistry (Figure 1a). The structure of HOA was characterized by ^1^H NMR, ^13^C NMR, and FT-IR analyses. The ^1^H NMR spectra of OA, ME, and HOA are presented in Figure 1b. The ^1^H NMR spectrum of OA exhibits a characteristic peak at 5.29 ppm, corresponding to the olefinic protons of the —C***H***=C***H***— bonds. In the spectrum of ME, the characteristic signal at 2.19 ppm is assigned to the thiol group (—S***H***), while the hydroxyl proton (—O***H***) appears near 4.86 ppm. However, the signals corresponding to —C***H***=C***H***— bonds are absent in the spectra of HOA, indicating that the olefinic feedstock has been completely converted. To further confirm this, the double-bond conversion was quantitatively evaluated by ^1^H NMR integral analysis. Taking the terminal methyl group (–CH_3_, δ = 0.86 ppm, 3H) as an internal reference, the integral ratio of the olefinic protons (–CH=CH–, δ ≈ 5.29 ppm) to the methyl protons was calculated for both OA and HOA. For OA, the ratio I_(5.29 ppm)_/I_(0.86 ppm)_ = 1.95/3 = 0.65, consistent with the theoretical value of 2/3. For HOA, the integral at 5.29 ppm was 0, yielding a double-bond conversion of approximately 100%. This result confirms that the thiol–ene click reaction proceeded with essentially complete conversion under the solvent-free UV-initiated conditions. Moreover, the HOA spectrum exhibits a characteristic carboxyl peak at approximately 12.1 ppm (—COO***H***) alongside a signal at 4.74 ppm attributed to the —O***H*** group, while the resonance at 2.60 ppm is assigned to the —C***H***—S— protons.

In the ^13^C NMR spectrum of the HOA monomer, the signal at 130.0 ppm corresponding to the olefinic carbon of the OA chain is absent. Meanwhile, new resonances emerged at 174.6 ppm, attributable to the carboxyl carbon, and at 45.8 ppm, assigned to the –CH–S– carbon (Figure S2). FTIR spectra of OA, ME, and HOA are presented in Figure S3. In the spectrum of HOA, the absorption band at 3011 cm^−1^, attributed to the C–H stretching of the olefinic C=C bond, is no longer observed. Concurrently, the band at 3478 cm^−1^ corresponding to the –OH group increases in intensity. The successful synthesis of HOA monomers via the thiol–ene click reaction is confirmed by these results.

To determine the carboxyl and hydroxyl groups in HOA monomers, the acid value of HOA was determined according to ASTM D1980-87, yielding a result of 145.2 ± 3.4 mg KOH/g, and the hydroxyl value was measured following ASTM D1957-86, giving a value of 137.82 ± 20.0 mg KOH/g. Additionally, thermogravimetric analysis (TGA) reveals that the initial decomposition temperature (*T*_5%_) of HOA is 257 °C (Figure S4). This temperature is markedly above the processing temperature (170 °C) employed for melt compounding, which confirms that HOA maintains its thermal stability during the entire dynamic vulcanization process.

### 3.2. Reaction Mechanism of PLA/HPUA Blends

Figure S5 displays torque versus mixing time profiles of PLA/HPUA blends. Upon PLA addition, the torque gradually decreases with mixing time. When HOA is introduced into the blending chamber, the torque drops immediately owing to its lubricating action. However, after the subsequent addition of HDI, the melt torque begins to increase as mixing proceeds and then gradually levels off, confirming the occurrence of a reaction between HOA and HDI. Furthermore, the final torque value increases as the total addition of (HOA + HDI) increases. This is likely attributed to the formation of more crosslinked products, as evidenced by the continuously increasing gel content observed in subsequent measurements (Table S1).

**Figure 2:**
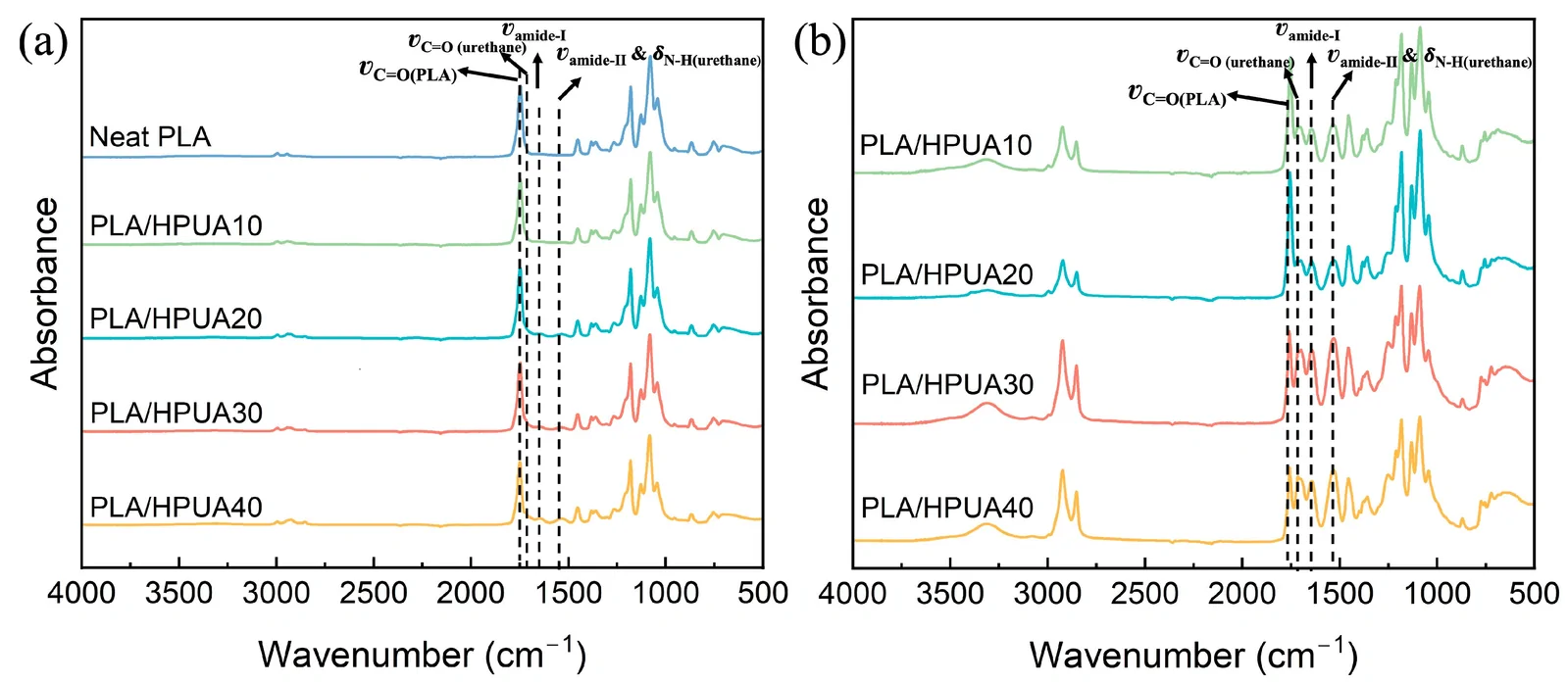
FTIR spectra of neat PLA and PLA/HPUA blends: (**a**) non-extracted and (**b**) the CHCl_3_-etching treatment.

To elucidate the reaction mechanisms during melt compounding, FTIR spectra of neat PLA and non-extracted PLA/HPUA blends are presented in Figure 2a. The characteristic absorption peak of the –NCO group of HDI at 2250 cm^−1^ is not detectable in any spectrum of the PLA/HPUA blends, indicating that the HDI had been completely consumed during the dynamic vulcanization. The FTIR spectrum of the PLA/HPUA blend exhibits characteristic peaks at 1716 cm^−1^ and 1643 cm^−1^, corresponding to the C=O stretching shoulder of the urethane band and the amide I band, respectively. The peak at 1533 cm^−1^ is attributable to the N-H bending/C-N stretching bands of the urethane bond as well as the amide II band. These results are consistent with the formation of a poly(urethane-co-amide) network via in situ polymerization of HOA and HDI. However, it should be noted that reactions between isocyanates and carboxylic acids at melt-processing temperatures can proceed through complex pathways, and the resulting network may also contain urea, acylurea, biuret, and allophanate linkages as side products [44,45]. The FTIR evidence presented here supports the formation of a crosslinked network but cannot definitively distinguish among these specific structural motifs.

To further confirm that the HPUA elastomer forms a crosslinked network structure, the PLA/HPUA blend was etched with chloroform to remove uncrosslinked components, such as free PLA, unreacted toughening monomers, and non-crosslinked HPUA products. The insoluble residue was then collected and used to determine the gel content of the PLA/HPUA blend (Table S1). The measured gel contents for the PLA/HPUA10, PLA/HPUA20, PLA/HPUA30, and PLA/HPUA40 blends were 10.2%, 21.8%, 28.9%, and 39.9%, respectively, which correspond closely to the feeding fractions of both HOA and HDI. The corresponding sol contents were calculated to be 89.8%, 78.2%, 71.1%, and 60.1%, respectively. Notably, the gel content of the PLA/HPUA20 blend is slightly higher than the total feed amount of HOA and HDI, which can be attributed to reactive compatibilization between the PLA matrix and the HPUA phase, leading to the formation of PLA-*b*-HPUA copolymers. Additionally, the dried insoluble residue is analyzed by FTIR spectroscopy (Figure 2b). All residues display characteristic absorptions associated with urethane and amide groups, providing additional evidence for the formation of a crosslinked poly(urethane-*co*-amide) (i.e., c-HPUA) network. Furthermore, at a low n_NCO,HDI_/n_COOH+OH,HOA_ ratio of 1.2:1, the gel content of the PLA blend is only 6.8%, markedly below the total feed fraction, indicating that crosslinking is incomplete because of the insufficient excess of –NCO groups. In contrast, when the ratio is increased to the optimal value of 1.6:1, the gel content of the PLA/HPUA20 blend slightly exceeds the feed level, confirming that the excess –NCO effectively drives the formation of a fully crosslinked network. The presence of the PLA ester C=O stretching band at 1750 cm^−1^ in these residues, indicates that PLA chains are incorporated into the crosslinked HPUA network.

To further investigate the structure of the crosslinked network, solid-state ^13^C NMR analysis is performed on the chloroform-insoluble residue of the PLA/HPUA20 blend (Figure S9). A resonance is observed at 159.4 ppm in the spectrum, corresponding to the urethane carbonyl (–O–C(=O)–NH–). In addition, a broad carbonyl signal appears between 174 and 170 ppm, which is assigned to the overlapping contributions from the ester carbonyl of PLA and the carbonyl groups of the amide moieties. The presence of the PLA ester carbonyl in the insoluble residue provides additional evidence that PLA chains are covalently incorporated into the crosslinked HPUA network.

**Figure 3:**
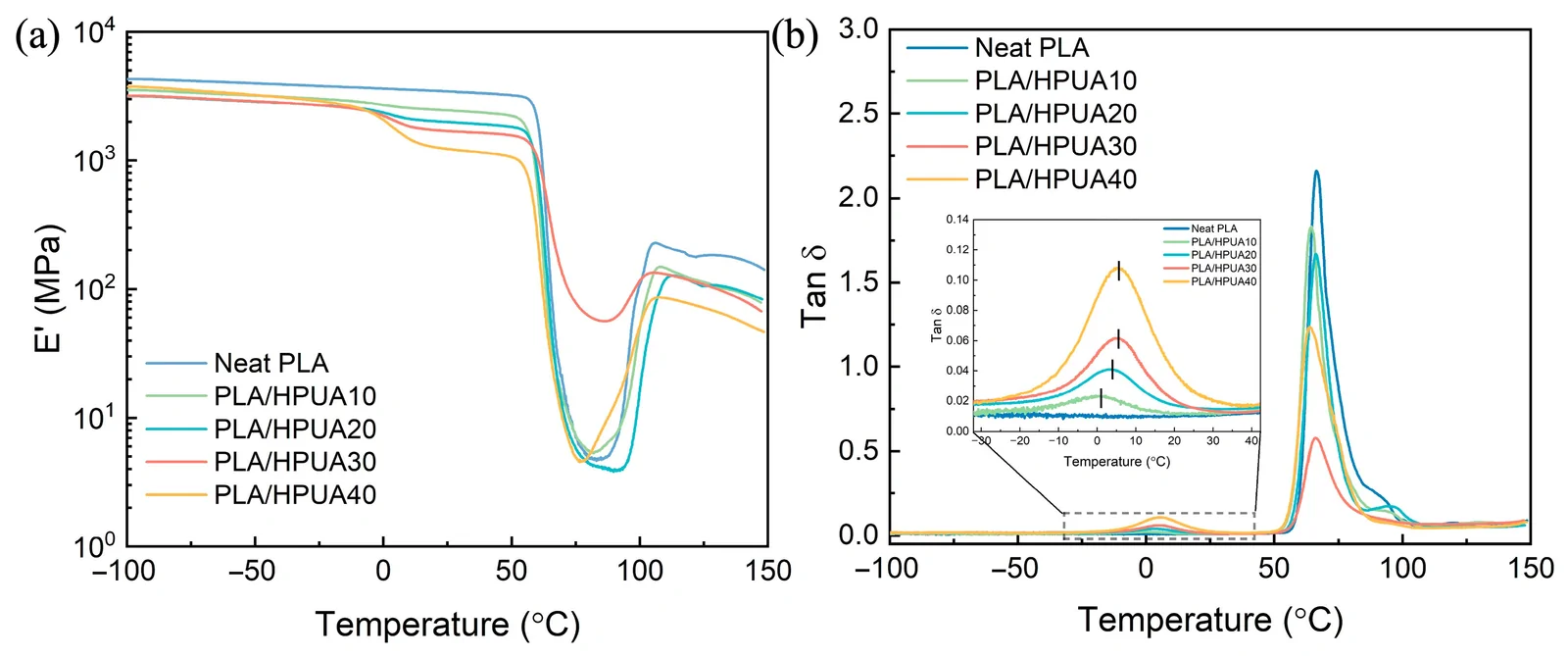
Storage modulus (**a**) and damping factor (**b**) for neat PLA and PLA/HPUA blends. The black lines in the inset indicate the peak positions (*T*_g,HPUA_) of the tan δ curves for each blend.

Figure 3 displays the storage modulus and damping factor of neat PLA and PLA/HPUA blends. Figure 3a shows that at temperatures below approximately 60 °C, the storage modulus decreased progressively as the HPUA content increased, which can be attributed to the in situ formation of the flexible HPUA phase. Compared to neat PLA, the PLA/HPUA blends exhibit a decrease in storage modulus within the temperature range of 0–20 °C, and the extent of this decrease increases with HPUA content, corresponding to the *T*_g_ of the HPUA phase (*T*_g,HPUA_) (Figure 3a). When the temperature exceeds 60 °C, the storage modulus of all samples drops dramatically, which is mainly attributed to the *T*_g_ of the PLA phase (*T*_g,PLA_). Figure 3b presents the damping factor as a function of temperature, where two distinct relaxation peaks corresponding to the HPUA and PLA domains are clearly observed. As the HPUA content increases, the *T*_g,HPUA_ of PLA/HPUA blends first rises and then levels off. At a HPUA content of 10%, the *T*_g,HPUA_ value is approximately −0.3 °C. When the HPUA content reaches 30%, the *T*_g,HPUA_ of the PLA/HPUA30 blend increases to 5.6 °C, which is 5.9 °C higher than that of the PLA/HPUA10 blend. The progressive increase in the tan δ peak height of the HPUA phase suggested an increasing volume fraction of this component, which correlated with the enhanced gel content of the HPUA phase as the total HOA/HDI loading was raised.

**Figure 4:**
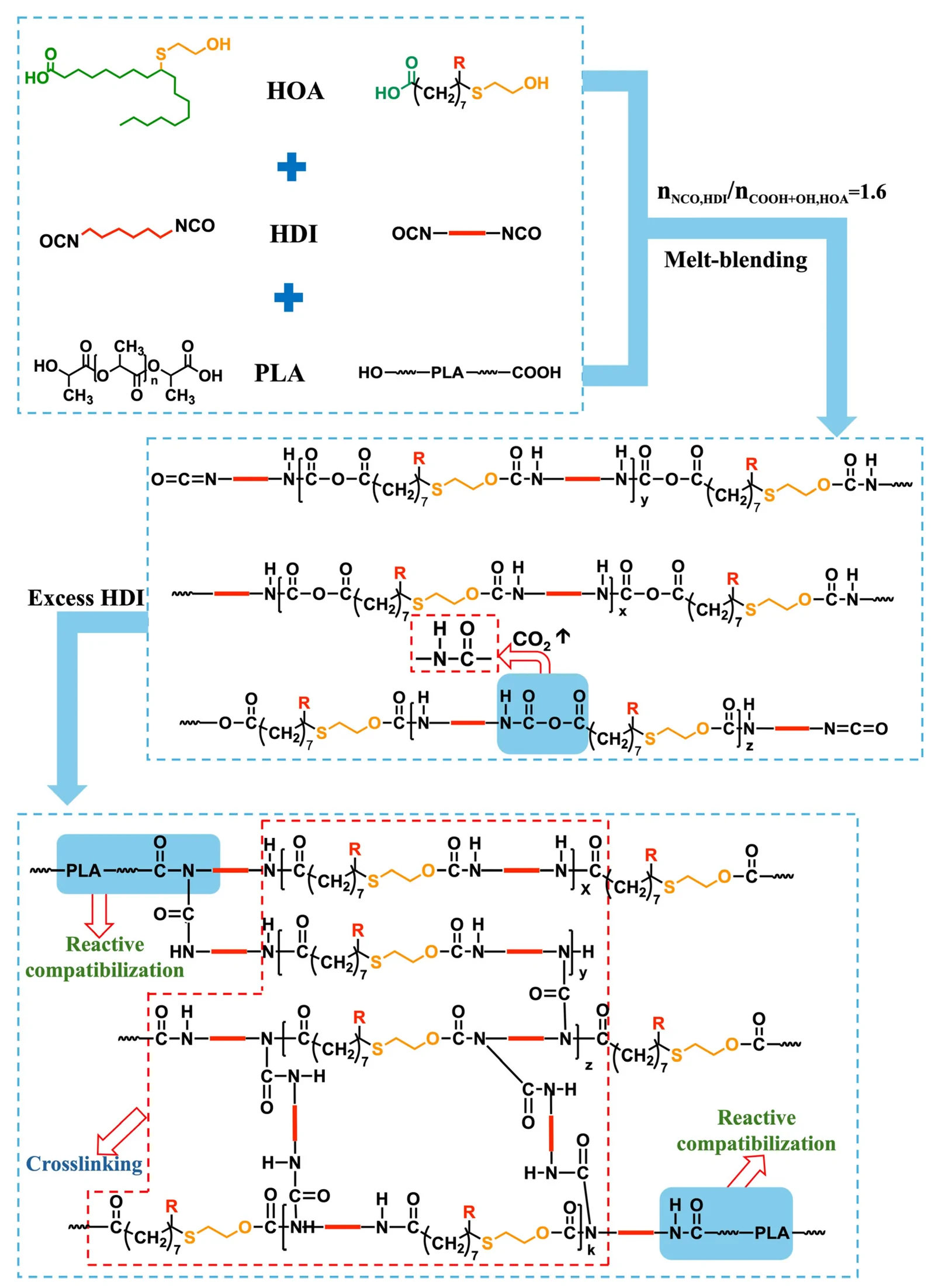
Schematic diagram illustrating the possible reaction mechanism of dynamic vulcanization of PLA with HOA and HDI during the melt blending process. The black arrow indicates CO_2_ released from the reaction between –NCO and –COOH groups.

Based on the results described above, a schematic of the proposed reaction mechanism during dynamic vulcanization is presented in Figure 4. During melt compounding, the –OH and –COOH groups of HOA can in situ react with the NCO groups of HDI, forming both urethane (–O–(C=O)–NH–) groups and amide (C(=O)−NH) ones. The active hydrogen atoms in the newly formed amide and urethane groups further reacted with the excess isocyanate groups that remained after complete consumption of the reactive groups of HOA, thereby generating a crosslinked HPUA elastomer within the PLA matrix. Furthermore, as the (HOA + HDI) content increases during the melt compounding process, more excess HDI is available to form a crosslinked HPUA network. The proposed reaction mechanism is supported by the observation that when the HDI-to-HOA molar ratio is 1.2:1, the gel content of the PLA/HPUA blend is only 6.8%. In addition, reactive compatibilization occurs between the isocyanate groups of the resulting HPUA elastomer and the terminal groups of PLA, further enhancing interfacial adhesion between the two phases.

### 3.3. Mechanical Properties of PLA/HPUA Blends

**Figure 5:**
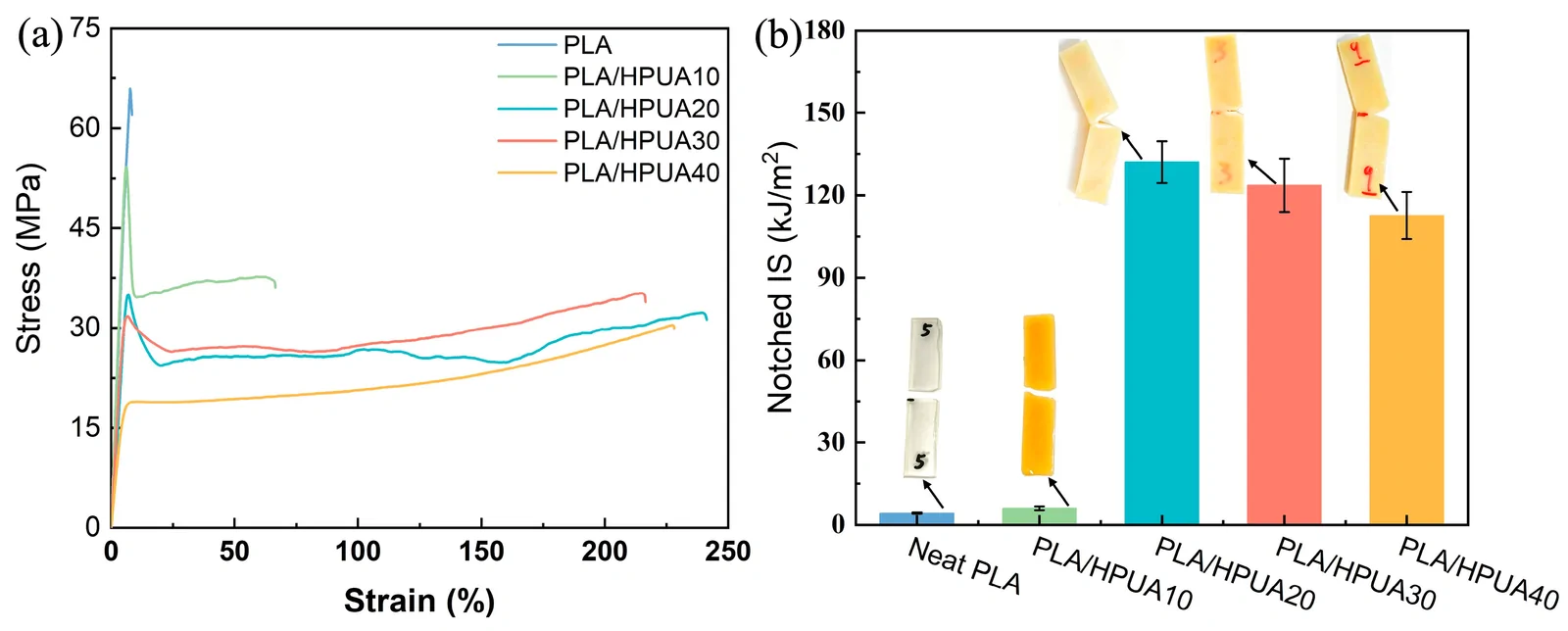
Stress–strain curves (**a**) and column plots of the Izod notched impact strength (IS) (**b**) of the neat PLA and PLA/HPUA blends.

Figure 5a presents the stress–strain curves of neat PLA and PLA/HPUA blends with various HPUA contents, and the corresponding mechanical property data are summarized in Table S2. Neat PLA exhibits rapid brittle fracture after a distinct yield without neck formation, showing a Young’s modulus of 3.6 GPa, a tensile strength of 65.7 MPa, and an elongation at break of approximately 8.5%. However, when HOA and HDI are incorporated into the PLA matrix for dynamic vulcanization, the resulting PLA/HPUA blends all exhibit highly ductile fracture behavior. When the HPUA content is 10 wt%, stable necking occurs followed by yielding without strain hardening, and the elongation at break reaches 70.8%. When the HPUA content is raised to 20 wt% or higher, strain hardening continues until ultimate failure, and the elongation at break of the blend exceeds 200%. However, as the HPUA content increases, both the Young’s modulus and the yield strength of the blends exhibit a gradual decline. Specifically, the Young’s modulus decreases from 3.6 GPa for neat PLA to 1.2 GPa for the PLA/HPUA40 blend, while the yield strength drops from 65.7 MPa to 30.0 MPa. Notably, At an HPUA content of 20 wt%, the elongation at break of the PLA/HPUA20 blend is 236.2%, which is 27.8 times that of neat PLA. When the HPUA content is increased to 40 wt%, the strain-hardening behavior becomes more pronounced with the absence of a clear yield point.

Figure 5b presents the notched Izod impact strength (IS) of the PLA/HPUA blends. The notched IS of neat PLA is only 4.3 kJ/m^2^. With the addition of only 10 wt% HPUA, the notched IS of the obtained PLA blend reaches merely 6.1 kJ/m^2^, showing no significant improvement over neat PLA. Notably, when the HPUA content exceeds 10 wt%, the notched IS of the PLA/HPUA blend shows a pronounced brittle-to-ductile transition. At an HPUA content of 20 wt%, the resulting PLA blend achieved optimal impact toughness, with a notched IS of 132.1 kJ/m^2^, which is 30.7 times that of neat PLA. Moreover, the test specimens do not fracture completely upon impact and display a distinct stress-whitening zone (see the inset in Figure 5b). Upon further increasing the HPUA content to 30 wt% or above, the notched IS of the blend decreases slightly but remains above 110 kJ/m^2^.

To distinguish the dynamic vulcanization effect from potential plasticization by HOA, DMA analysis was employed as a direct probe. If HOA acted as a plasticizer for the PLA matrix, *T*_g,PLA_ would be expected to decrease significantly. However, the *T*_g,PLA_ values of all PLA/HPUA blends showed no significant change (64.4–66.2 °C) compared to neat PLA (66.3 °C) (Table S1), indicating that HOA is consumed during the dynamic vulcanization reaction rather than persisting as a free plasticizer. This is further supported by the internal control provided by the PLA/HPUA20-1.2 blend (gel content = 6.8%, notched IS = 12.6 kJ/m^2^), which contains the same amount of HOA but lacks sufficient crosslinking, showing negligible toughening compared to the PLA/HPUA20 blend (gel content = 21.8%, notched IS = 132.1 kJ/m^2^).

### 3.4. Morphological Evolution of PLA/HPUA Blends

Scanning electron microscopy (SEM) images of cryo-fractured surfaces of PLA/HPUA blends are shown in Figure S6. The fracture surfaces of all PLA/HPUA blends reveal that the dispersed HPUA particles are hardly discernible from the PLA matrix, with no obvious phase boundaries or interfacial gaps, indicating strong interfacial adhesion between the PLA matrix and the HPUA phase. Notably, at an HPUA content of 20 wt%, the fracture surface becomes more homogeneous and flatter (Figure S6b). Upon further increasing the HPUA content to 30 wt% or beyond (Figure S6c,d), the cryo-fractured surface appears more rugged, featuring numerous coarse wrinkles.

**Figure 6:**
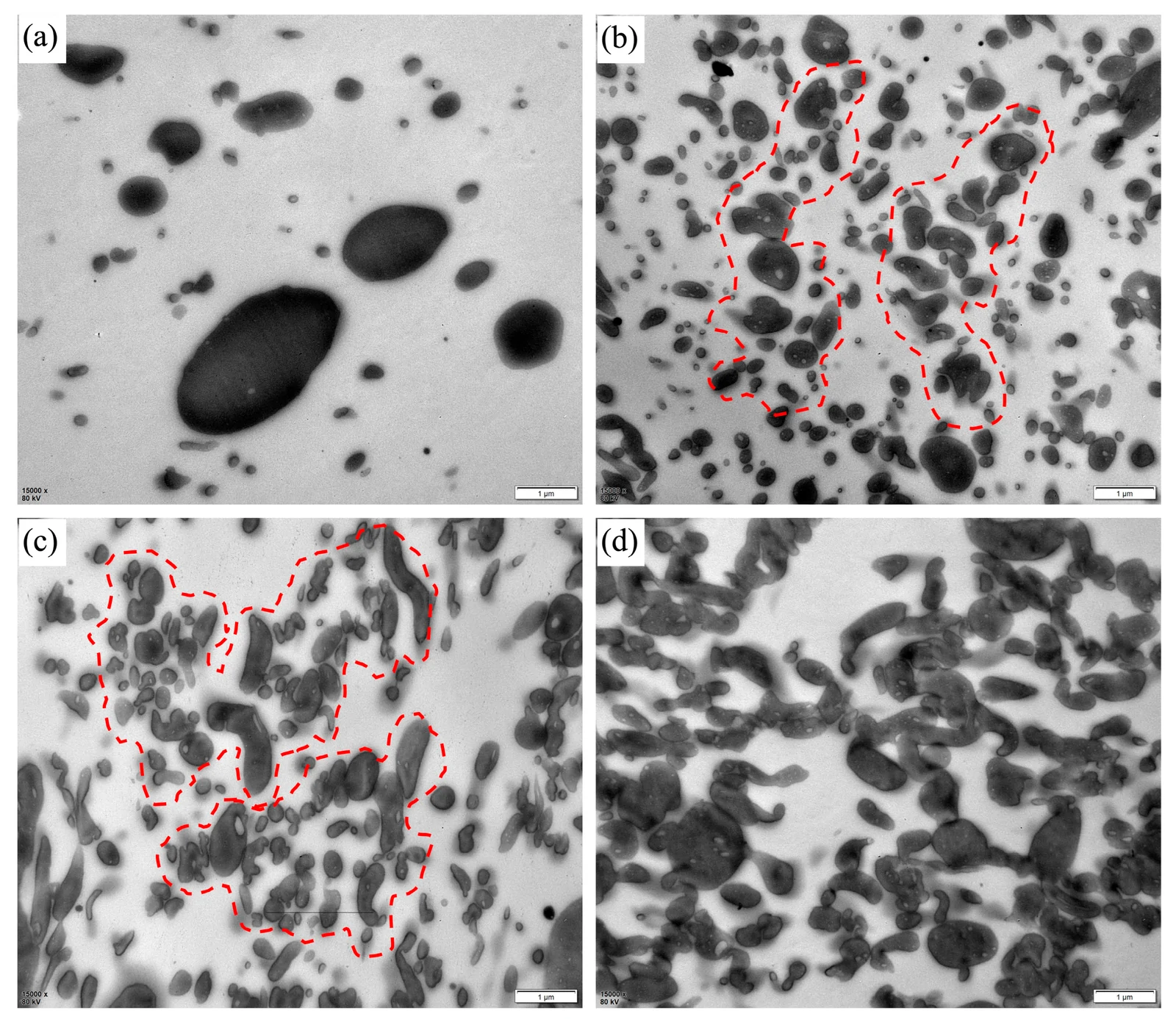
RuO_4_-stained TEM micrographs of PLA blends: (**a**) PLA/HPUA10, (**b**) PLA/HPUA20, (**c**) PLA/HPUA30, and (**d**) PLA/HPUA40.

To reveal the detailed phase morphology of the PLA/HPUA blends, Figure 6 presents their TEM images after staining with RuO_4_. When the HPUA content is 10 wt%, the blend exhibits a typical sea-island phase structure (Figure 6a), wherein dark-colored HPUA particles are randomly distributed throughout the continuous PLA matrix, which corresponded to its lower impact toughness. As the HPUA content is raised to 20 wt% and 30 wt%, the aggregation phenomenon becomes more pronounced, with some HPUA particles forming highly irregular pseudo-network structures (Figure 6b,c). When the HPUA content reaches 40 wt%, these cluster-like aggregates transformed into a bi-continuous network, accompanied by an increase in the size of the HPUA aggregate phase within the PLA/HPUA40 blend (Figure 6d).

To further confirm the phase continuity described above, 1.0 mm thick sheets of neat PLA and the PLA/HPUA blends were subjected to chloroform extraction to selectively dissolve the PLA phase. Owing to the loss of structural support from the continuous PLA phase, both neat PLA and the PLA/HPUA10 composite samples completely collapsed following 1.5 h of immersion, as depicted in Figure S7. But after the same treatment, the PLA/HPUA20 blend shows obvious swelling and crumpling yet still maintained relatively good self-supporting behavior, indicating that the HPUA phase retained a partially continuous structure. When the HPUA content is increased to 30 wt% or higher, the extracted blend samples preserved almost complete structural integrity, evidencing the establishment of a bi-continuous structure. Thus, the solvent extraction data corroborate well with the aforementioned TEM observations.

**Figure 7:**
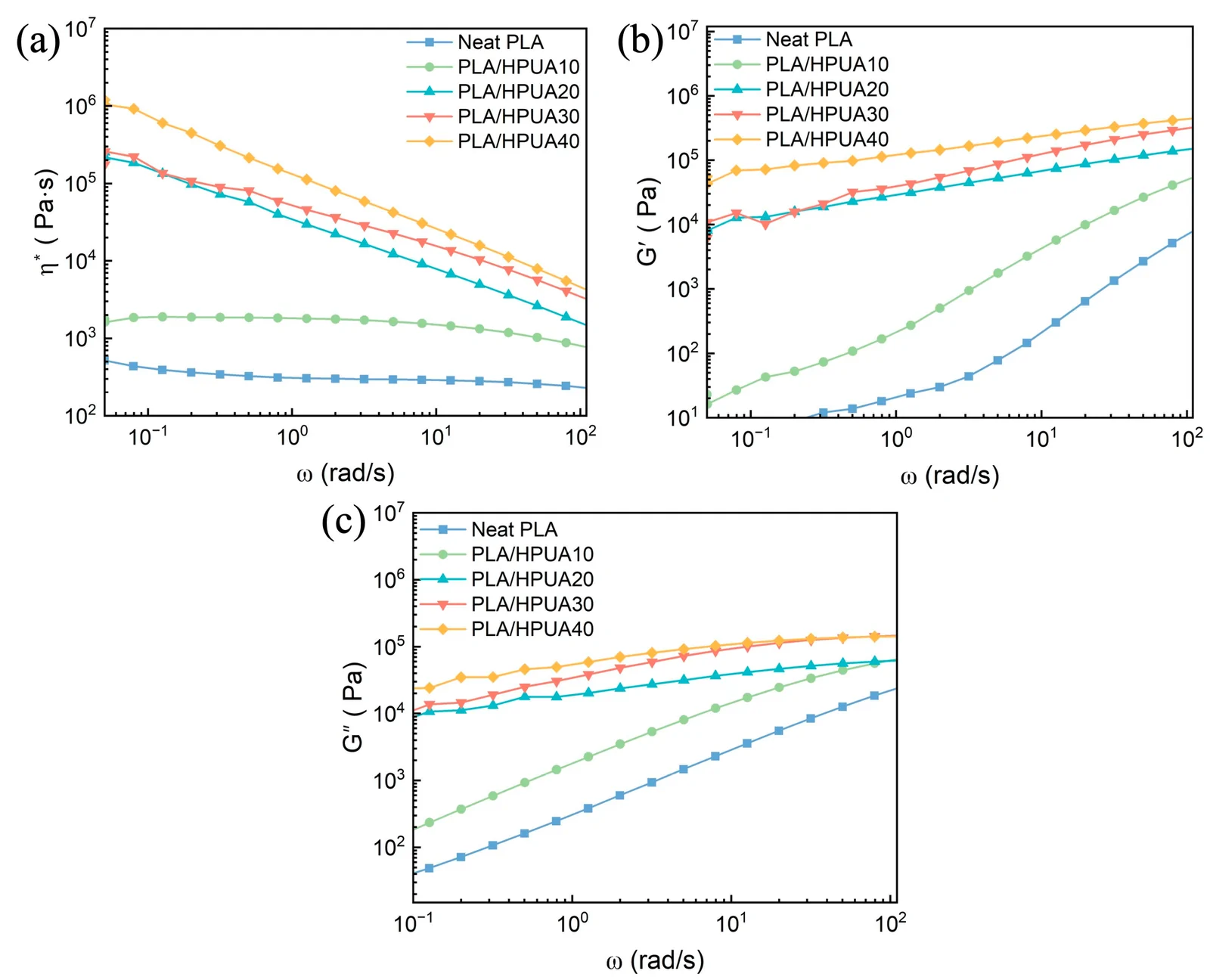
Dynamic rheological properties versus angular frequency at 180 °C for neat PLA and PLA/HPUA blends: (**a**) complex viscosity (ƞ*); (**b**) storage modulus (G′); (**c**) loss modulus (G″).

The morphological evolution from sea-island to bi-continuous structure can be understood through percolation theory. As the HPUA content increases, the volume fraction of the HPUA phase approaches and exceeds the percolation threshold, at which point the dispersed HPUA domains interconnect to form a continuous network throughout the PLA matrix. This transition is corroborated by both the selective extraction results (Figure S7), where the HPUA network gains self-supporting capability at ≥20 wt%, and the rheological data (Figure 7), where the emergence of a low-frequency G′ plateau indicates the formation of a percolated network structure.

### 3.5. Rheological Properties of PLA/HPUA Blends

Rheological testing provides a sensitive tool for examining the phase structure of polymer blends. The evolution of complex viscosity (η*), storage modulus (G′), and loss modulus (G″) with angular frequency for the neat PLA and PLA/HPUA blends is presented in Figure 7. Neat PLA displays typical Newtonian behavior across the tested frequency range (Figure 7a). The η* values of the PLA/HPUA blends increase monotonically with increasing HPUA content. The Newtonian plateau remains discernible when the HPUA content reaches 10 wt%. However, upon increasing the HPUA content to 20 wt% or higher, these blends exhibit pronounced shear-thinning behavior, accompanied by a further significant increase in η* across the entire frequency range. At low frequencies, η* even exceeds that of neat PLA by more than two orders of magnitude. This result is likely attributable to the denser crosslinked network of the HPUA phase and the formation of a more perfect bi-continuous morphology under these conditions. The dependence of storage modulus (G′) and loss modulus (G″) on angular frequency (ω) is shown in Figure 7b,c. Neat PLA exhibits typical terminal flow behavior characteristic of viscoelastic liquids, where approximately G′ ∝ ω^2^ and G″ ∝ ω. As the HPUA content increases, both G′ and G″ of the PLA/HPUA blend exhibit a progressively upward trend, with G′ remaining higher than G″ across the entire frequency range. When the HPUA content reaches 20 wt% or higher, the terminal slopes of both moduli exhibit a weak dependence on angular frequency, indicating a more pronounced solid-like response. Such non-terminal behavior is attributed to crosslinked chains or entangled networks formed in the PLA/HPUA melt.

To verify the structural stability of the PLA/HPUA blends during frequency-sweep measurements at 180 °C, we conducted an isothermal time-sweep test on the PLA/HPUA20 blend at a frequency of 1 rad/s for 30 min. The time-sweep data (Figure S10) show no monotonic increase in G′ or η* over the 30 min duration, confirming that no post-crosslinking occurs during testing. A gradual and monotonic decrease in G′, G″, and η* is observed over the test period. Although minor thermal effects cannot be entirely excluded, the absence of an accelerating modulus loss suggests that significant thermal degradation do not occur at the test temperature. Since the frequency sweep (0.05–200 rad/s) requires only 440 s to complete, the changes within this window are modest and do not affect the interpretation of the frequency sweep curves.

### 3.6. Impact-Toughening Mechanism

**Figure 8:**
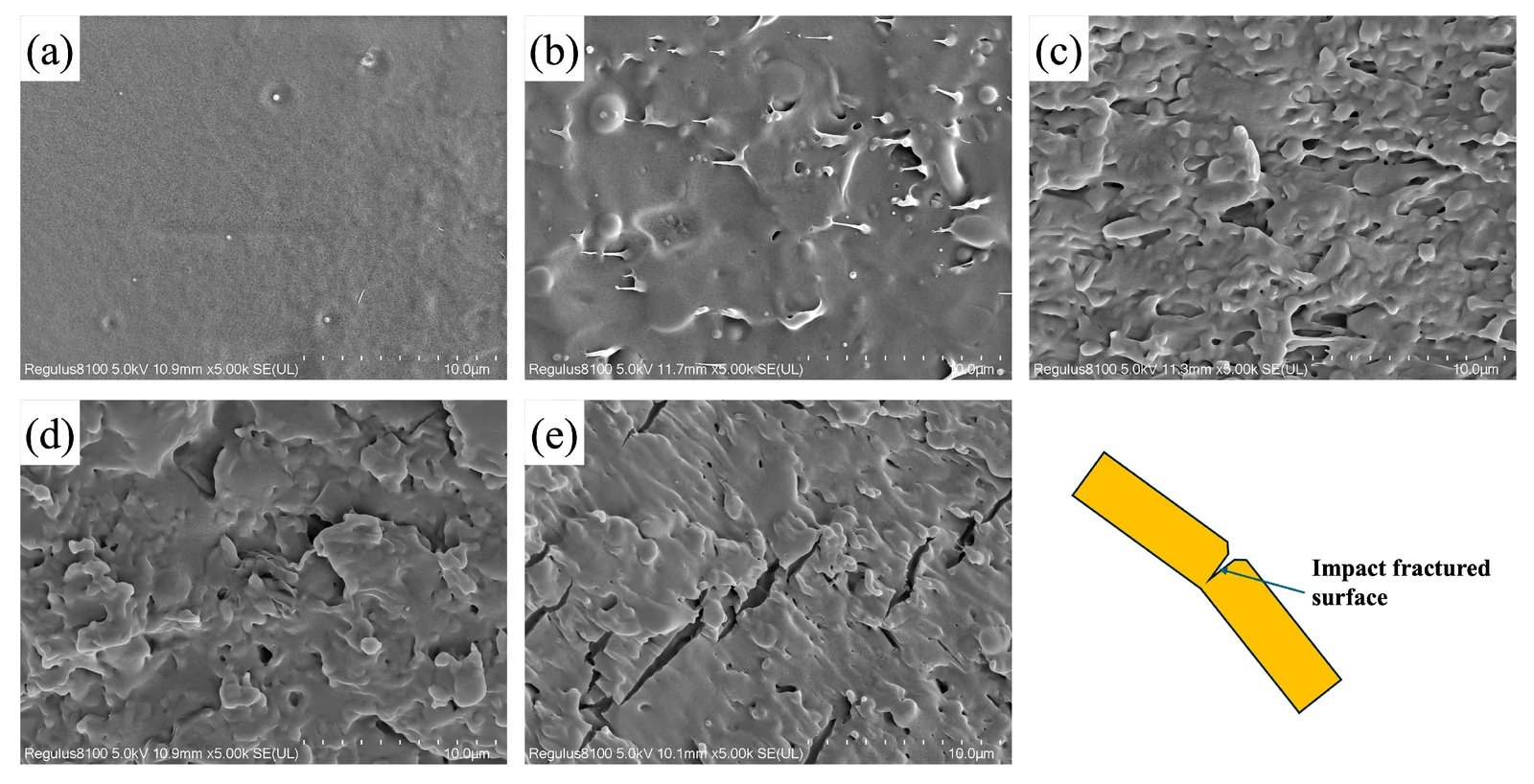
SEM micrographs of impact-fractured surfaces of neat PLA and various PLA/HPUA blends: (**a**) neat PLA; (**b**) PLA/HPUA10; (**c**) PLA/HPUA20; (**d**) PLA/HPUA30; and (**e**) PLA/HPUA40.

Figure 8 shows SEM micrographs of impact-fractured surfaces of neat PLA and various PLA/HPUA blends. The fracture surface of neat PLA appears smooth and featureless (Figure 8a). In addition to the existence of some smooth, micrometer-sized pits (Figure 8b), a similar morphology is observed for the PLA/HPUA10 blend, which is consistent with its extremely low impact strength. At an HPUA content of 20 wt%, the blend exhibits a rougher fracture surface (Figure 8c). The dispersed particles are scarcely distinguishable from the PLA matrix, and no obvious debonding is observed, indicating good interfacial adhesion between the toughening phase and the PLA matrix. In addition, plastic deformation of the matrix is clearly observed, accompanied by the presence of many voids, which is consistent with the excellent impact resistance of the blend. When the HPUA content reaches 30 wt% or higher (Figure 8d,e), the deformation of the entire matrix further evolves into oriented plastic deformation.

In elastomer-toughened polymer systems, it is generally accepted that microvoiding is the critical step that triggers matrix yielding, a process that is accompanied by substantial energy dissipation during impact testing [18]. Microvoiding generally arises from either internal cavitation inside the elastomer particles or debonding at the particle–matrix interface. The former is favored by strong interfacial adhesion and low cavitation resistance, while the latter takes place when the elastomer–matrix adhesion is suitable [7,8].

**Figure 9:**
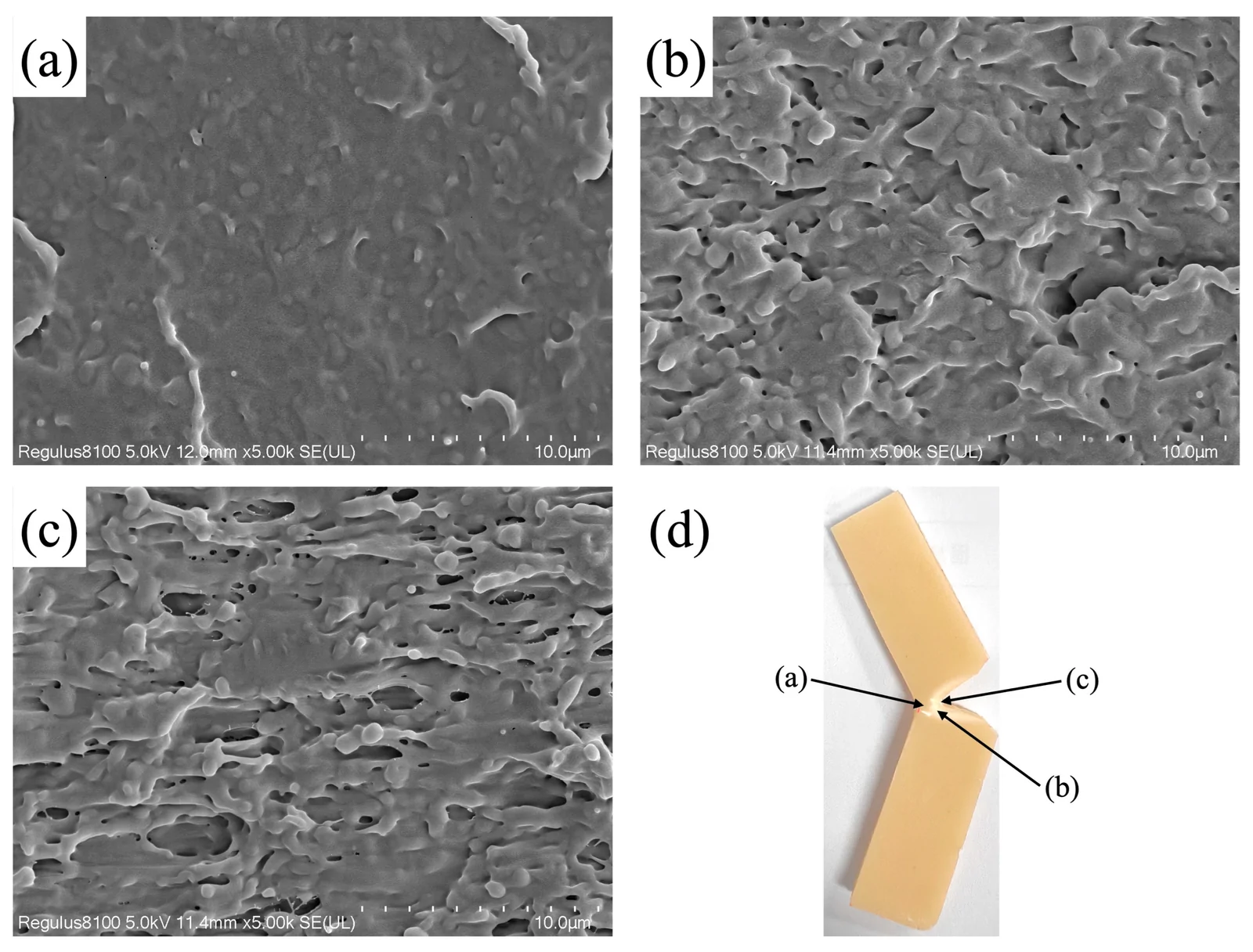
SEM images for the surfaces at different zones (**a**–**c**) of the unbroken joint of impact PLA/HPUA20 sample bar as schematically indicated in part (**d**).

The impact-toughening mechanism is further explored by observing the fracture surfaces of the non-fractured regions in the PLA/HPUA20 impact specimens using SEM (Figure 9d). The morphology of the blend remains largely unchanged in regions far from the crack (Figure 9a). Within the stress-whitened region, extensive irregular cavities are observed (Figure 9b). Concurrently, the matrix surrounding the cavities show signs of orientation and deformation, resulting from internal cavitation of the HPUA domains under impact stress. In the region near the crack, both the deformation and orientation of the cavities and the matrix rapidly developed into highly oriented thread-like shapes (Figure 9c). Therefore, under impact loading, the super-tough PLA/HPUA blend follows a toughening mechanism involving internal cavitation of the HPUA elastomer phase, which triggers matrix shear yielding and plastic deformation, contributing remarkably to energy dissipation during impact.

To further elucidate the micromechanical deformation process of PLA/HPUA blends, we have performed TEM analysis of the subfracture region immediately beneath the surface of the stress-whitening zone of the PLA/HPUA20 impact test specimens (Figure S8). The TEM micrographs reveal that some microvoids are located inside the HPUA particles rather than at the particle–matrix interface. This provides direct evidence that the internal cavitation of HPUA domains occurs during impact testing. The internal cavitation is favored by the strong interfacial adhesion between the HPUA phase and the PLA matrix, which prevents interfacial debonding and forces the stress concentration to be released through cavitation within the lower-modulus HPUA domains.

## 4. Conclusions

In this work, hydroxylated oleic acid (HOA) was first synthesized from oleic acid via UV-initiated thiol–ene click chemistry. Subsequently, a dynamic self-vulcanization strategy that involved using bifunctional monomers to toughen PLA, HOA and an excess of hexamethylene diisocyanate (HDI) were used as the toughening monomers. During melt blending, a crosslinked flexible poly(urethane-*co*-amide) (c-HPUA)-toughening phase was formed via in situ polymerization between HOA and HDI, while the interfacial compatibility between the HPUA phase and the PLA matrix significantly improved. When the HPUA content reached 20 wt% or higher, the blend exhibited a bi-continuous morphology, and the notched IS of the PLA/HPUA blend exhibited a clear brittle-to-ductile transition. At an HPUA content of 20 wt%, the resulting PLA blend exhibited optimal impact toughness, with an elongation at break of 236.2% and a notched IS of 132.1 kJ/m^2^, which was 30.7 times that of neat PLA. When the HPUA content was further increased to 30 wt% or higher, the notched IS of the blend decreased slightly but remained above 110 kJ/m^2^. The primary toughening mechanism was identified as internal cavitation of the HPUA domains, which in turn triggered yielding of the surrounding PLA matrix. This study provides valuable insights into the design of high-performance PLA materials using a dynamic self-vulcanization strategy with bifunctional monomers.

## Data Availability

The raw data supporting the conclusions of this article will be made available by the authors on request.

## Supplementary Materials

The following supporting information can be downloaded at: https://www.mdpi.com/article/10.3390/polym18161981/s1, Figure S1: Stress–strain curves (a) and column plots of the Izod notched impact strength (IS) (b) of the neat PLA and PLA/HPUA blends with different n_NCO,HDI_/n_COOH+OH,HOA_ ratios.; Figure S2: ^13^C NMR spectra of OA, ME, and HOA; Figure S3: FTIR spectra of OA, ME and HOA; Figure S4: TGA and DTG curves of HOA; Figure S5: Torque versus mixing time profiles of PLA/HPUA blends; Figure S6: SEM images of cryo-fractured surfaces of PLA/HPUA blends: (a) PLA/HPUA10, (b) PLA/HPUA20, (c) PLA/HPUA30, and (d) PLA/HPUA40; Figure S7: Photographs of 1-mm-thick sheet specimens of neat PLA and PLA/HPUA blends before and after 1.5 h of extraction in chloroform; Figure S8: TEM micrograph of the stress-whitening zone immediately beneath the impact-fractured surface of the PLA/HPUA20 blend specimen; Figure S9: Solid-state ^13^C NMR spectrum of the chloroform-insoluble residue from the PLA/HPUA20 blend; Figure S10: Storage modulus (G′), loss modulus (G″), and complex viscosity (η*) of the PLA/HPUA20 blend as a function of time (30 min) during an isothermal time-sweep test at 1 rad/s, 1% strain, and 180 °C; Table S1: The gel content, crosslinking density and glass transition temperatures (*T*_g_) of each component of the PLA blends; Table S2: Mechanical data of neat PLA and PLA blends.
