# Supplementary material for: Constructing Bi-Continuous Poly(urethane-co-amide) Networks from Hydroxylated Oleic Acid via Dynamic Self-Vulcanization for Super-Toughened Polylactic Acid Blends
Source: Polymers (Basel). 2026 Aug 14;18(16):1981. doi: 10.3390/polym18161981 (PMC13516864; doi:10.3390/polym18161981)
Supplement: Supplementary file 1 [file polymers-18-01981-s001.zip › polymers-4471197-supplementary.pdf]

Supporting information

Constructing Bi-continuous Poly(urethane-*co*-amide) Networks from Hydroxylated Oleic Acid via Dynamic Self-vulcanization for Super-toughened Polylactic acid blends

Dongmei Xie*, Xiaodi Mao, Hongyu Li, Xudong Chen, Yuting Li, Hongzhi Liu*

School of Materials Science and Engineering, NingboTech University, Ningbo 315100, Zhejiang Province, China

*Corresponding authors, **E-mail**: xiedongmei@nbt.edu.cn (Dr. Dongmei Xie); hzliu@iccas.ac.cn (Prof. Hongzhi Liu)

Table S1. The gel content, crosslinking density and glass transition temperatures (T_g) of each component of the PLA blends.

Sample	Ratio	Gel content (%)	$T_{g,HPUA}$ (°C)	$T_{g,PLA}$ (°C)
Neat PLA	-	--	--	66.3
PLA/HPUA20-1.2	1.2:1	6.8±1.4	-5.3	62.8
PLA/HPUA20-1.4	1.4:1	14.9±3.8	1.3	62.2
PLA/HPUA 20-1.8	1.8:1	24.2±0.7	3.6	63.4
PLA/HPUA10	1.6:1	10.2±1.1	-0.3	64.4
PLA/HPUA20	1.6:1	21.8±1.3	3.0	66.2
PLA/HPUA30	1.6:1	28.9±0.4	5.6	65.9
PLA/HPUA40	1.6:1	39.9±0.3	5.2	63.7

Note: $T_{g,polyamide}$ and $T_{g,PLA}$ were determined by DMA.

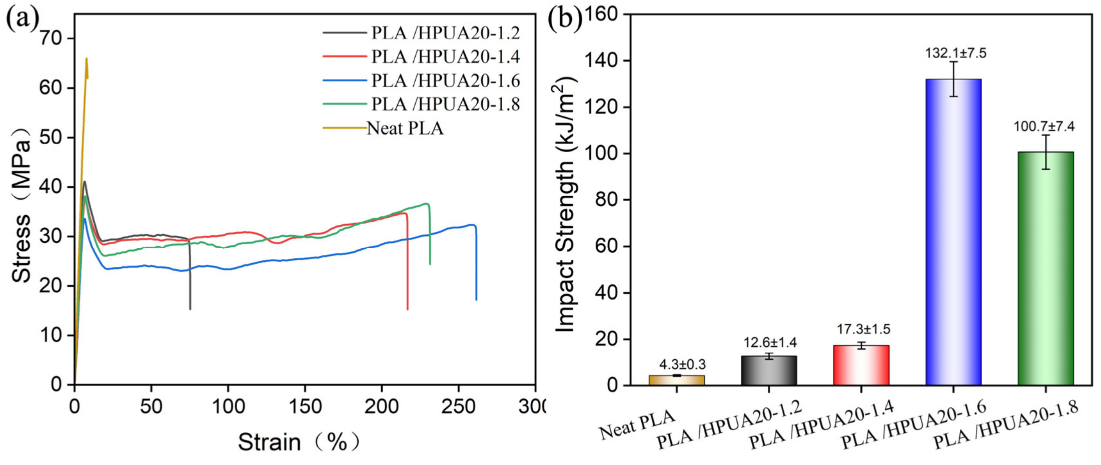

FigureS1. Stress–strain curves (a) and column plots of the Izod notched impact strength (IS) (b) of the neat PLA and PLA/HPUA blends with different $n_{NCO,HDI}/n_{COOH+OH,HOA}$ ratios.

Table S2 Mechanical data of neat PLA and PLA blends.

Sample	Ratio	Young's modulus (GPa)	Tensile strength (MPa)	yield strength (MPa)	Elongation at break (%)	Notched IS (kJ/m^2)
Neat PLA	-	3.6±0.5	65.7±2.1	65.7±2.1	8.5±0.5	4.3±0.3
PLA/HPUA20-1.2	1.2:1	2.7±0.2	40.9±0.9	40.9±0.9	70.8±3.6	12.6±1.4
PLA/HPUA20-1.4	1.4:1	2.1±0.3	35.3±2.2	35.3±2.2	183.4±34.3	17.3±1.5
PLA/HPUA 20-1.8	1.8:1	2.9±0.4	38.5±1.3	38.0±0.7	253.9±39.1	100.7± 7.4
PLA/HPUA10	1.6:1	2.7±0.3	53.7±1.0	53.7±1.0	70.8±9.9	6.1±0.7
PLA/HPUA20	1.6:1	2.4±0.2	35.0±0.7	34.6±0.6	236.2±16.8	132.1±7.5
PLA/HPUA30	1.6:1	2.0±0.3	33.7±3.3	30.8±0.7	205.0±24.7	123.6±9.7
PLA/HPUA40	1.6:1	1.2±0.1	30.0±2.5	-	228.1±23.4	112.7±8.6

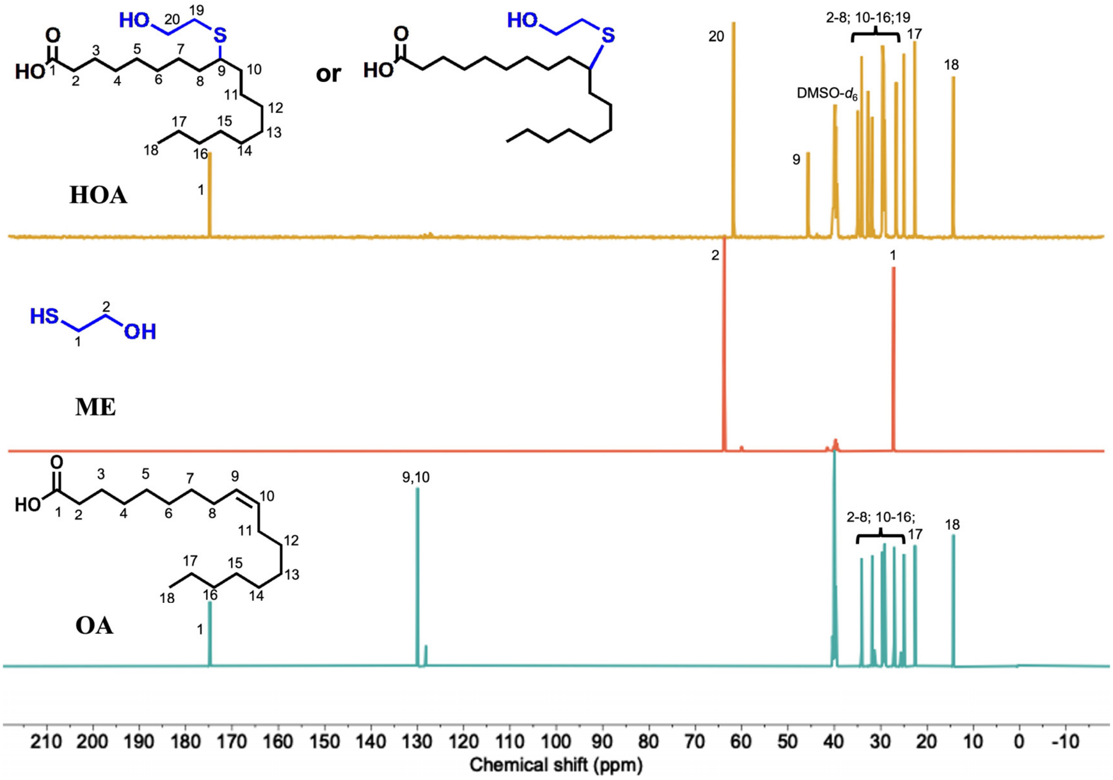

Figure S2. ^{13}C NMR spectra of OA, ME, and HOA.

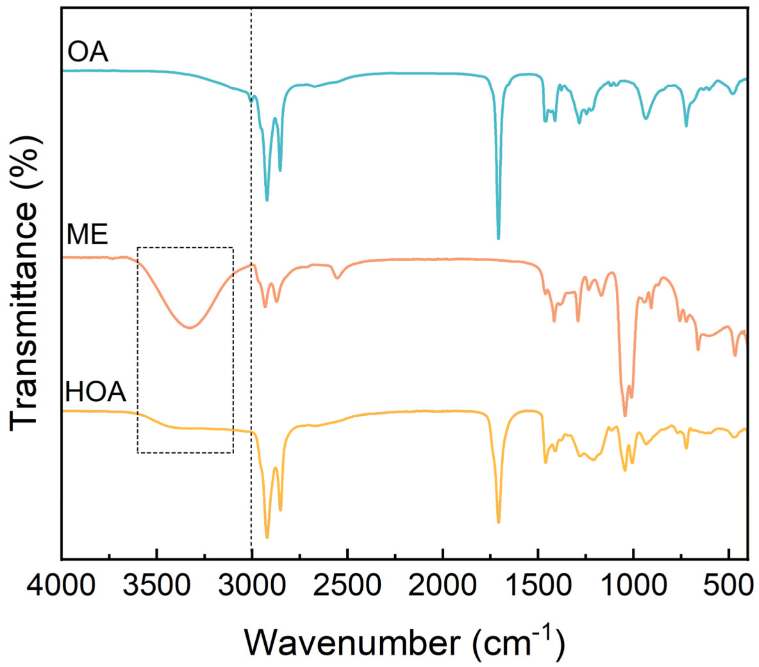

Figure S3. FTIR spectra of OA, ME and HOA.

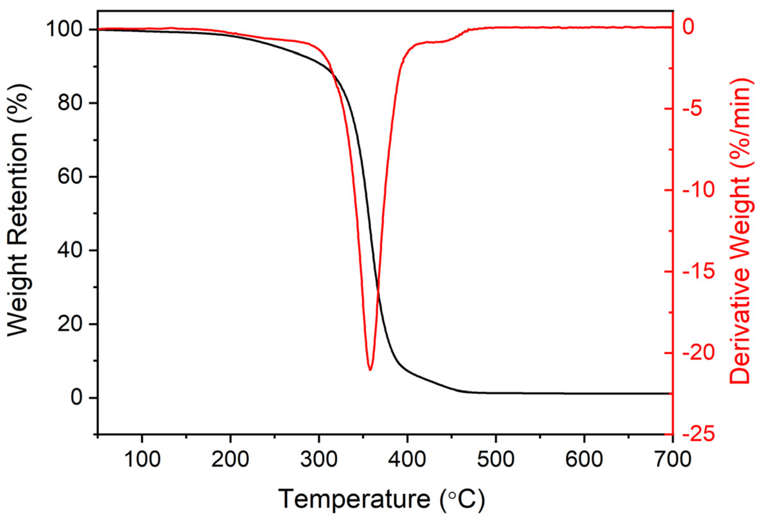

Figure S4. TGA and DTG curves of HOA.

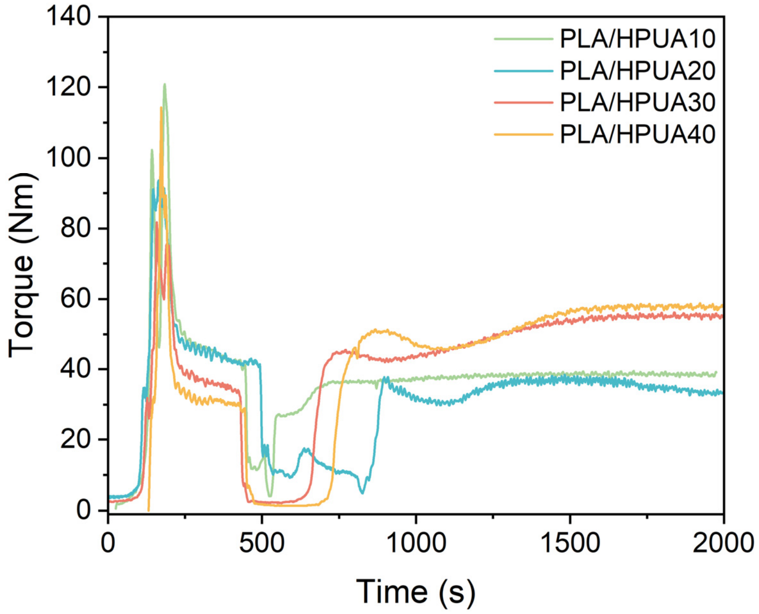

Figure S5. Torque versus mixing time profiles of PLA/HPUA blends.

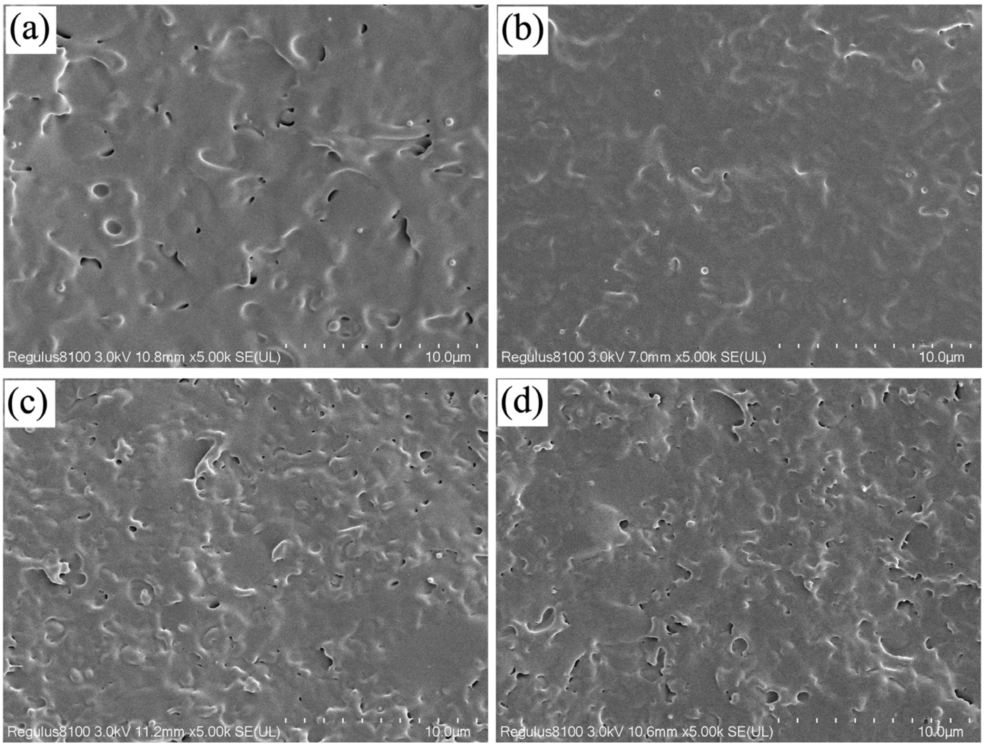

Figure S6. SEM images of cryo-fractured surfaces of PLA/HPUA blends: (a) PLA/HPUA10, (b) PLA/HPUA20, (c) PLA/HPUA30, and (d) PLA/HPUA40.

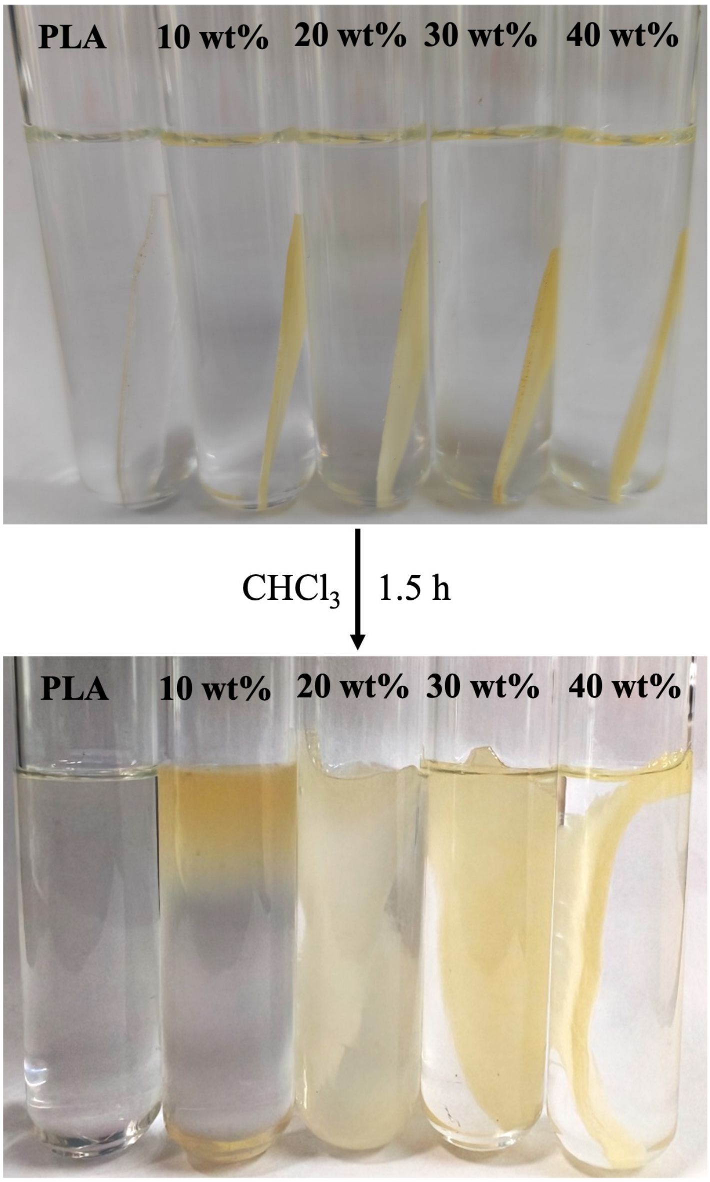

Figure S7. Photographs of 1-mm-thick sheet specimens of neat PLA and PLA/HPUA blends before and after 1.5 h of extraction in chloroform.

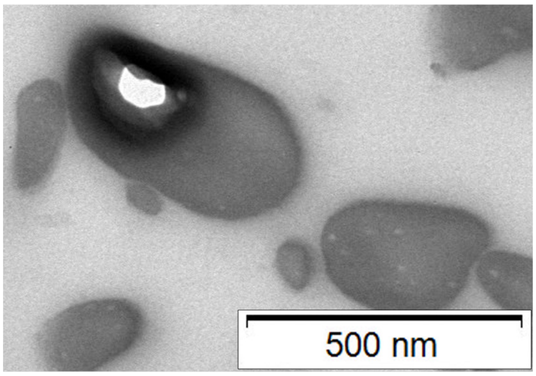

Figure S8. TEM micrograph of the stress-whitening zone immediately beneath the impact-fractured surface of the PLA/HPUA20 blend specimen.

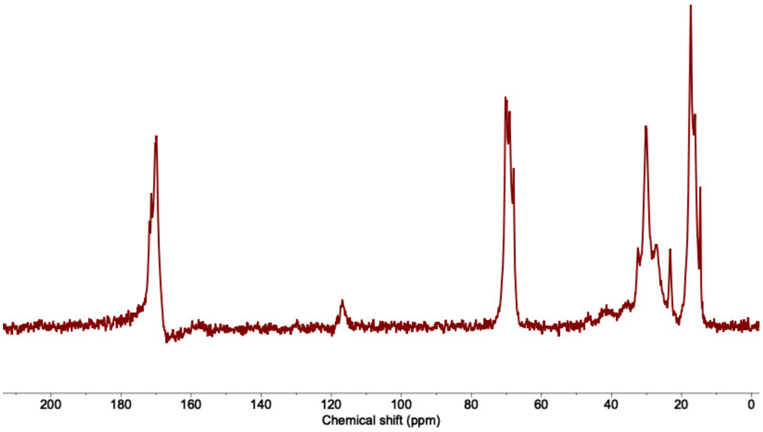

Figure S9. Solid-state ^{13}C NMR spectrum of the chloroform-insoluble residue from the PLA/HPUA20 blend.

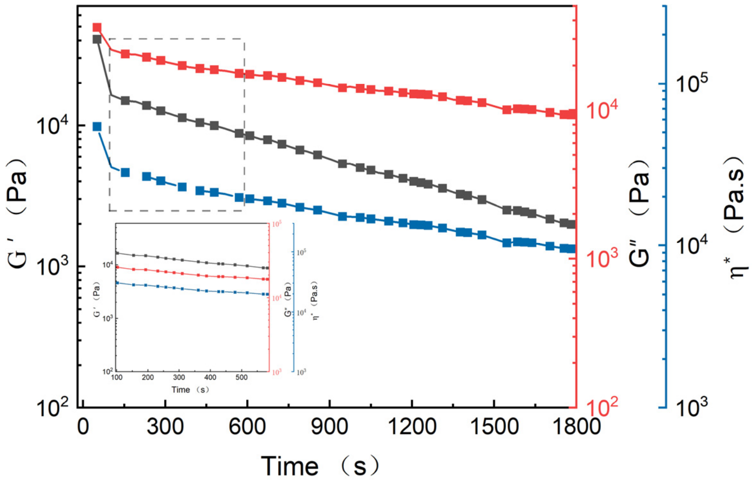

Figure S10. Storage modulus (G′), loss modulus (G″), and complex viscosity (η*) of

the PLA/HPUA20 blend as a function of time (30 min) during an isothermal time-sweep test at 1 rad/s, 1% strain, and 180 °C.
